# Effect of Canagliflozin Pretreatment on the Efficacy of Insulin Therapy to Rescue Type 1 Diabetes-Related Bone Fragility in Male Mice

**DOI:** 10.1007/s00223-026-01486-x

**Published:** 2026-01-31

**Authors:** Jeffry S. Nyman, R. Clay Bunn, Sasidhar Uppuganti, Elizabeth M. Hennen, Plaban Mishra, Philip D. Ray, Anthony Garcia Mendez, Evangelia Kalaitzoglou, John L. Fowlkes

**Affiliations:** 1 Department of Orthopaedic Surgery, Vanderbilt University Medical Center, 1215 21st, Ave. S., Suite 4200, Nashville, TN 37232 USA; 2 Department of Biomedical Engineering, Vanderbilt University, 5824 Stevenson Center, Nashville, TN 37232 USA; 3 United States Department of Veterans Affairs, Tennessee Valley Healthcare System, 1310 24th Ave. S., Nashville, TN 37212 USA; 4 Department of Pediatrics and Barnstable Brown Diabetes Center, University of Kentucky, 830 S. Limestone St., Lexington, KY 40536 USA

**Keywords:** Canagliflozin, Advanced glycation end-products, Bone quality, Hyperglycemia

## Abstract

Humans with type 1 diabetes (T1D) have a higher-than-expected fracture risk, and bone is weak in mouse models of T1D. Since hyperglycemia is thought to deleteriously affect tissue-level sensitivity of insulin action, we hypothesized that glucose-lowering by canagliflozin (Cana) improves the ability of insulin treatment to rescue the T1D-related decrease in bone strength. To test this hypothesis, male DBA/2 mice with and without streptozotocin (STZ)-induced hyperglycemia at 8 weeks of age were fed a diet with and without Cana (50 ppm) at 10 weeks of age and then the T1D mice, regardless of placebo diet or Cana diet, were treated at 14 weeks of age with a subcutaneous implant (Palm) that does or does not elute insulin. Non-diabetic (ND) mice and T1D mice were euthanized at 22 weeks of age following weekly measurements of body mass and non-fasting blood glucose. Cana pre-treatment significantly lowered circulating glucose, but not to non-diabetic levels. It did not affect the ability of insulin therapy to reduce glucose. However, pre-treatment significantly affected serum markers of bone resorption (CTX) and bone formation in ways that favored lower CTX across the 3 glucose groups (ND-Palm, T1D-Palm, and T1D-Insulin) and P1NP levels in T1D-Insulin mice that were consistent with P1NP levels in ND mice. Cana pre-treatment did not affect bone strength (compression of lumbar vertebra and bending of femur mid-diaphysis) nor the ability of insulin therapy to improve bone strength. Cana and glucose group did not affect advanced glycation end-products in the organic matrix of bone, despite glycated hemoglobin A1c being much higher in the T1D mice. Cana and glucose group both independently affected crack initiation toughness such that insulin rescued the T1D-related decline of cortical bone to resist crack growth and Cana modestly added to this effect. Lastly, T1D lowered bound water in the bone matrix and insulin treatment partially reversed the decline. Thus, glycemic control prior to insulin treatment does not appear to affect the ability of insulin to prevent bone fragility in T1D mice.

**Supplementary Information:**

The online version contains supplementary material available at 10.1007/s00223-026-01486-x.

## Introduction

The skeletal system has emerged as a target tissue of diabetes-mediated damage in what is now called diabetic bone disease (DBD) [1]. DBD is a complication that underpins the well-known propensity of humans with type 1 diabetes (T1D) experiencing a fragility fracture [2–4]. Although fracture risk is higher in those with T1D than those without diabetes across the human lifespan [2], low areal bone mineral density (aBMD) in T1D insufficiently explains the elevated fracture risk at the hip and upper extremity [5], suggesting that other T1D-related factors may contribute to bone fragility [6]. Moreover, clinical studies suggest a negative association between glycemic control, as determined by glycated hemoglobin A1c (HbA1c), and aBMD in adults [3] and children [7, 8] with T1D, but such an association was not observed in adults with long-standing T1D (duration ≥ 50 year) that was well controlled with exogenous insulin [9]. Indeed, poor glycemic control and long duration are independent risk factors of fractures in T1D [10]. Despite improvements in the administration of insulin to those with T1D, thereby more consistently maintaining healthy levels of circulating glucose, fractures in those with T1D remain a clinical problem with multiple possibilities for how T1D causes bone fragility beyond low bone mass [11].

Rodent models of T1D provide translational and pre-clinical opportunities by which to study possible factors that may contribute to DBD. Exogenous insulin therapy in T1D rodents improves many of the abnormalities that occur in DBD, as well as decreasing peripheral glucose levels [12–15]. Insulin therapy has been shown to enhance bone formation and improve fracture healing in T1D rodents [16–18], implicating their role in promoting osteogenesis and bone formation. Another possible factor that may contribute to DBD is hyperglycemia. However, in a mouse model of T1D, we found that improving systemic glucose concentrations to near-normoglycemia by an inhibitor of the renal sodium-dependent glucose co-transporter type 2 (SGLT2i) did not prevent the diabetes-related bone deficits and poor fracture resistance [14, 19]. Thus, insulin signaling in osteogenic cells is likely important to maintaining sufficient bone strength [20].

Long-term glycemic control is associated with protection from many complications of T1D in humans [21]; however, the impact of prior hyperglycemia on the response of the skeleton to insulin therapy has not been studied in humans or rodents. Nevertheless, in vitro studies of bone marrow stromal cells from T1D rats and control rats suggest insulin deficiency and subsequent hyperglycemia alone may diminish expression of the insulin receptor [22]. In a mouse model of T1D, hyperglycemia decreased insulin receptor substrate 1 (IRS-1), an important mediator of both insulin and insulin-like growth factor 1 (IGF-1) signaling [23, 24]. Peripheral insulin resistance can develop in T1D [25] meaning insulin therapy becomes less effective in controlling glucose over time. These observations suggest prior exposure to hyperglycemia (i.e., under-insulinized or poorly controlled T1D) might impact the efficacy of insulin to improve or protect any organ, including bone, from damage.

Based on prior research in rodent models of T1D, improving hyperglycemia alone is likely not sufficient to prevent DBD [13, 19]. As such, we postulate that the severity of hyperglycemia impacts the skeletal response to exogenous insulin with respect to preventing and treating bone fragility in T1D. To investigate this supposition, we treated T1D mice (DBA/2J) with or without canagliflozin, an FDA-approved SGLT2i for lowering glucose in type 2 diabetes, to decrease peripheral glucose levels for 1 month before treating the T1D mice with exogenous insulin therapy. Since injecting streptozotocin (STZ) into 7-week-old, male DBA/2 J reproducibly induces sufficient beta cell death, T1D mice develop hypoinsulinemia and hyperglycemia within 3 weeks as well as experience bone weakness within 10 weeks of the STZ injections [26]. Such a model represents what happens when children develop T1D prior to peak bone mass but without the autoimmune origins of the disease [27]. Therefore, 2.5 weeks after the last STZ injection, Cana pre-treatment was started to lower but not normalize blood glucose; and insulin was started at ~ 14-weeks of age when mice typically approach peak bone mass. Insulin treatment lasted for 8 weeks to increase the likelihood of rescuing the deficit in bone structure and bone strength.

## Materials and Methods

### Study Design

Six-week-old, male, DBA/2 J mice were obtained from The Jackson Laboratory (Bar Harbor, Maine) and were acclimated to the vivarium for one week. Approaching 7 weeks of age, mice were injected intraperitoneally with STZ at 40 mg/kg (*n* = 44) or citrate buffer (*n* = 20) one time per day for five consecutive days. Non-fasting blood glucose (BG) was measured after ten days following the last STZ or buffer injection to confirm hyperglycemia (BG > 400 mg/dl) and the onset of type 1 diabetes (T1D). BG was monitored weekly using a < 1 μl drop of blood from the tail vein placed on an AlphaTrak3 (Zoetis, Kalamazoo, MI) test strip. At ~ 10 weeks of age, diabetic (T1D) and non-diabetic (ND) mice were randomized to receive a commercial rodent diet (2918 from Inotiv, Indianapolis, IN) containing canagliflozin (MedChemExpress, Monmouth Junction, NJ) at 50 ppm (T1D-Cana, *n* = 24) or 62.5 ppm (ND + Cana, *n* = 10) or the same diet without Cana (T1D-Diet, *n* = 24; ND-Diet, *n* = 10) for four weeks. The lower concentration of Cana in the diet offsets the higher food intake of T1D mice compared to non-diabetic mice. Body mass, blood glucose (BG), and food consumption were measured weekly. At ~ 14 weeks of age, all mice were fed the 2918 rodent diet (Inotiv, Indianapolis, IN) without canagliflozin ad libitum. The T1D mice that received Cana or the diet alone were then implanted with sustained-release insulin (Ins) implants called LinBit (T1D-Diet-Ins, *n* = 10; T1D-Cana-Ins, *n* = 14) or with blank palmitic acid micro-crystal (Palm) implants (T1D-Diet-Palm, *n* = 14; T1D-Cana-Palm, *n* = 10). Non-diabetic mice were implanted with Palm implants (ND + Veh + Palm, *n* = 10; ND-Cana-Palm, *n* = 10). All implants were inserted and replaced as needed based on manufacturer’s recommendations (LinShin Canada, Inc., Toronto, Ontario) such that most mice received 3 implants based on body mass being close to 25 g (i.e., 2 for 20 g plus additional pellet(s) for each 5 g increase in weight). LinBit releases insulin at ~ 0.1 units/day/implant. Additional Ins implants were implanted in three mice from the T1D-Cana-Ins group and six mice from the T1D-Chow-Ins group approximately two weeks after initial implant placement due non-fasting BG levels greater than 450 mg/dl in these mice during the weekly check. Two additional mice from the T1D-Cana-Ins group received additional LinBit pellet approximately six weeks after the initial implant placement due to a non-fasting BG greater than 450 mg/dl.

After euthanasia by isoflurane inhalation and decapitation, blood and bones were collected. The blood was spun to extract serum. The left femur and L6 vertebral body (VB) were stored in phosphate buffered saline (PBS). The right femur was stored in 70% ethanol. All procedures followed a protocol approved by IACUC at the University of Kentucky.

### Serum Analysis of Metabolism

Collecting whole blood into serum and EDTA plasma tubes, clotting occurred on ice for approximately two hours followed by centrifugation to separate serum. An aliquot of EDTA-treated whole blood was removed and stored for HbA1c assay. Remaining EDTA-treated whole blood was centrifuged to separate plasma. Serum, plasma, and whole blood samples were stored at −20 °C until assayed. N-terminal Propeptide of Type 1 Procollagen (P1NP) was measured using a rat/mouse P1NP ELISA kit (Euroimmune US, Mountain Lakes, NJ Cat# AC-33F1). C-terminal telopeptides of type 1 collagen (CTX-I, Euroimmune US Cat#AC-06F1) was measured with RatLaps (CTX-I) EIA. Glycated hemoglobin was measured in EDTA-treated whole blood with an enzymatic mouse Hemoglobin A1c assay kit (Crystal Chem, Elk Grove Village, IL, USA, Cat # 80310).

### Micro-Computed Tomography (Μct) Evaluations of Femurs and Vertebral Body

After securing each left femur in a Scanco specimen tube holder (outer diameter = 6 mm; length = 30 mm) and filling the tube with phosphate buffered saline (PBS), a 2 mm segment centered at the mid-diaphysis and a 2.7 mm segment above the growth plate were scanned using an ex vivo μCT scanner (μCT50, SCANCO Medical AG, Brüttisellen, Switzerland) and typical scan parameters (Table S1) as described in our previous publications [14, 19]. The reconstructed images had an isotropic voxel size of 6 μm. Upon fitting contours to the periosteal surfaces of the cortex or drawing contours within the endosteal boundary of the metaphysis (451 slices starting 210 μm from the growth plate), the Scanco scripts for evaluations of cortical bone and trabecular bone, respectively, were applied to each region of interest using a Gaussian image noise filter and a global threshold for segmentation to obtain standard measurements of cortical structure and trabecular architecture. Including a calibration of hydroxyapatite (HA) phantom, which is scanned weekly, and beam hardening correction, the scripts measured the mean tissue mineral density (TMD), which is HA-calibrated attenuation of all bone voxels (post segmentation) with a peel of 2 surface voxels to minimize partial volume effects.

The micro-notched right femurs and the 5th lumbar (L5) vertebra (VB) were imaged by μCT and evaluated in a similar way but different scan and image segmentation parameters (Table S1). For these femurs, the creation of the micro-notch on the posterior side and the angle of the micro-notched was determined as previously described [28]. The evaluation of the trabecular bone in the L5 centrum was like the evaluation distal femur metaphysis and specifically followed our previously published study [29].

### Micro Finite Element Analysis (μFEA) of the L5 Vertebral Body

To create a three-dimensional finite element (FE) model of each μCT scan of the L5 VB, a circle with a constant radius of 1.25 mm (area = 49.3 mm^2^), which was larger than the VB, was copied into each image slice between the end plates and positioned to transect posterior elements that did not bear load in the compression test (i.e., removed the transverse and spinous processes). This VOI was segmented like the evaluation of the trabecular bone within the L5 VB (Table S1), thereby capturing the cortical shell that surrounds the trabecular.

Upon converting segmented bone voxels to 8-node hexahedral elements to generate the FE model, element-wise strain values were calculated for high-friction, axial compression loading of each VB (Problem number 33 in FE-software v1.13, Scanco Medical AG, Brüttisellen, Switzerland). Specifically, caudal nodes were constrained in the x-, y- and z-directions, while the cranial nodes were constrained in x- and y-direction with a negative displacement in the z-direction to achieve an apparent strain of 1%. Elastic modulus (E_t_ in MPa) was distributed into 45–60 materials based on incremental bins of TMD (increment of ~41 mg·HA/cm^3^ between 315.9 mg·HA/cm^3^ and 2,787.3 mg·HA/cm^3^) such that E_t_ = 0.1127 x TMD_*i*_^1.746^ [30], where TMD_*i*_ is the median TMD of bin *i*. A Poisson’s ratio of 0.3 was assigned to all elements. The estimated failure load of each VB in compression was the reaction force that caused 2% of the bone volume to exceed an equivalent strain of 0.007.

### Measurement of Bound Water in Femur by ^1^H Nuclear Magnetic Resonance (NMR) Relaxometry

The volume fraction of bound water (BW) was quantified by ^1^H nuclear magnetic resonance (NMR) relaxometry [31, 32] to determine if T1D and treatment affects tissue hydration, a matrix-sensitive determinant of bone mechanical properties [33, 34]. Since the right femurs were stored in 70% ethanol, they were rehydrated prior to NMR. Rehydration involved placing each femur in 15 ml tubes and filling the tubes with successive solutions of ethanol: 50% ethanol for 3 days and 25% ethanol for 3 days, both at 4 °C. Finally, each femur was immersed in phosphate buffer saline (PBS) at pH 7 (supplemented with 0.01% sodium azide) after thorough rinsing of both the bone and its tube with PBS. They were stored in 4 °C for 3 days prior to NMR analysis and then transferred to −20 °C after NMR analysis. Following rehydration and prior to micro-notching, the femur and a microsphere of water as a reference volume were sealed within a custom loop-gap-style radiofrequency coil and inserted into a 4.7 T horizontal bore magnet (Varian, Palo Alto, CA). Using a Carr–Purcell–Meiboom–Gill (CPMG) pulse sequence (10,000 echoes at 100 μs echo spacing), ^1^H NMR signals were acquired and processed as previously described [35]. The integrated area of the bound water peak within the T_2_ spectrum (Fig. S1) was converted to volume of water based on the integrated area of the microsphere of water (21.2 μl) and then divided by volume of bone (estimated using the Archimedes’ principle).

### Mechanical Testing of Femurs and Vertebral Body

Like our previous bone studies involving T1D mice [14, 19, 26], each hydrated left femur was loaded-to-failure at 3 mm/min in three-point bending using a mechanical testing system (Instron DynaMight 8841, Norwood, MA) with a 100 N load cell (Honeywell, OH, Model no. 060-C863-02). The diaphysis was oriented anterior side facing down (tension) and medial side facing forward. For all tests, the span was 8 mm. Each rehydrated right femur was loaded at 0.5 mm/min in three-point bending until the crack propagated from the micro-notch through the cortical bone causing failure (Fig. S2). By reducing the loading rate, there is more time for toughening mechanisms (e.g., collagen unwinding and crack deflection at lamellar interfaces) to resist crack growth. The posterior side with the micro-notched femur faced down (medial forward), and the span was 4 times the anterior-posterior width (determined by caliper) rounded to the nearest 0.1 mm. Force vs. displacement data was recorded from load cell and linear variable differential transducer. Further details and the determination of the mechanical properties can be found in our previous publications [28, 31, 36]. Likewise, the mechanical testing of the L6 VB to determine compressive strength (ultimate load) followed the methods in previous publications [29, 37].

### Analysis of Fluorescent Advanced Glycation End-Products (fAGEs) and Collagen Crosslinks

The entire broken left femur (following three-point bending tests) were demineralized in 20% EDTA at 4 °C for 5 weeks and then hydrolyzed in 6 N HCl at 110 °C for 22 h. After hydrolysis, the hydrochloric acid was evaporated using a SpeedVac Concentrator System (Thermo Fisher Scientific, Waltham, MA) with a cold trap. Next, each hydrolysate was suspended in HPLC-grade water and centrifuged at 15,000 g and 4 °C for 20 min.

Pentosidine (PEN), pyridinoline (PYD), and deoxypyridinoline crosslinks were measured using a high-performance liquid chromatography (HPLC) system (Agilent 1260 Infinity, Agilent Technologies, Santa Clara, CA) equipped with a Spherisorb 4.6 × 150 mm column (Waters Co, Milford, MA) as previously described [37]. The non-enzymatic and mature enzymatic crosslinks were calculated based on the standard curve that was derived from the chromatogram (Fig. S3) of the standard at different concentrations and normalized to collagen levels measured in the same samples [38].

Following our published methods [37, 38], aliquots of hydrolyzed samples and quinine sulphate standards were dissolved in 0.1 M H_2_SO_4_. Fluorescence was measured at λex/λem = 370 nm/440 nm using BioTek Synergy H1 microplate reader (BioTek Instruments, Winooski, VT). fAGE levels were calculated based on the standard curve and normalized to collagen levels in the same samples.

Collagen levels were based on measurements of hydroxyproline (Hyp) levels using a colorimetric assay [39]. Performed in 96-well plates following the addition of the oxidizer and Ehrlich's solution to Hyp standards and the hydrolysate samples, the color was developed upon the incubation of the plates at 65 °C for 20 min and consequent rapid cooling. The measurements were taken at 550 nm using the same microplate reader.

### Statistical Analysis

We used two-way analysis of variance (ANOVA) to determine if each outcome measure depended on Cana pre-treatment (No Cana or Cana), glucose group (ND-Palm, T1D-Palm, and T1D-Insulin), or their interaction (Prism v10.4., GraphPad Software, LLC, Boston, MA, USA). If the residuals of the ANOVA were heteroscedastic (Spearman’s test for each predicted outcome vs. its absolute residual) and/or not Gaussian (Anderson–Darling normality test for normality), we used ANOVA of Aligned Rank Transform from the ART library in R (R version 4.4.3, Vienna, Austria). When glucose group or the interaction term had a significant effect (*p*-value < 0.05) on a property, 3 pairwise comparisons (ND-Palm vs. T1D-Palm, ND-Palm vs. T1D-Ins, and T1D-Palm vs. T1D-Ins within each Cana group; 2 separate families) were tested for significance (family-wise *α* = 0.05) using the Holm-Šídák’s adjustment of the p-values or using the Dunn’s adjustment of the *p*-values. The latter non-parametric test was used when the values of each group did not pass the Shapiro–Wilk test for normality and/or their standard deviations were heterogeneous according to the Brown-Forsythe test. Likewise, these same pairwise adjustments under the same conditions of homogeneous variance and normality were used to determine if Cana pre-treatment had a significant effect within each glucose group (ND-Palm-No Cana vs. ND-Palm-Cana, T1D-Palm-No Cana vs. T1D-Palm-Cana, and T1D-Ins-No Cana vs. T1D-Ins-Cana; 1 family). To determine if body mass and circulating blood glucose depended on the age of the animal, Cana pre-treatment, insulin therapy, and their interactions, we used mixed-effects models (Prism v10.4., GraphPad Software, LLC, Boston, MA, USA) in which these weekly measurements were repeated but could be missing for certain groups at a certain age (categorical variable).

## Results

### Pre-treating Mice with Canagliflozin (Cana) to Lower Blood Glucose did Not Enhance the Effectiveness of Insulin Therapy to Normalize Blood Glucose Levels, Improve Survivability, and Incur Weight Gains in T1D mice

**Fig. 1:**
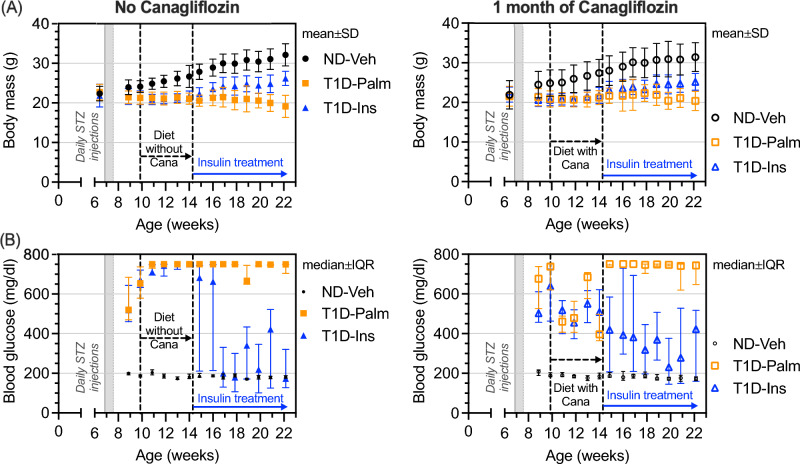
Change in body mass and blood glucose as a function of age. **A** Starting at the same body weight, mice lost body mass if they received the STZ injections to induce type 1 diabetes, while non-diabetic mice gained body mass as they matured. Late treatment with insulin partially recovered the weight gain, regardless if they received canagliflozin (Cana) pre-treatment. **B** As intended, the STZ injections causes severe hyperglycemia. Cana treatment ameliorated this hyperglycemia (bottom right), while insulin treatment further reduced non-fasting blood glucose levels

Between 10 weeks of age (start of Cana treatment) and 22 weeks of age (euthanasia), both Cana pre-treatment and insulin therapy lowered circulating glucose (Fig. 1A), but the effect of insulin did not depend on the prior treatment with Cana (interaction *p*-value = 0.2377 in Table 1). Likewise, Cana pre-treatment did not affect the ability of insulin to lower blood glucose when analyzing these weekly measurements from 15 weeks of age (i.e., start of Insulin treatment) to 22 weeks of age (Table 1). Only insulin treatment affected body mass (Fig. 1B) between 10 weeks and 22 week of age and between 15 and 22 weeks of age (Table 1). In untreated mice, STZ-T1D resulted in significant elevations in non-fasting circulating blood glucose levels reaching the limit of the glucometer and reducing the variance (Fig. 1B). Canagliflozin pre-treatment decreased blood glucose levels below 600 mg/dl during the one month of treatment, but only the insulin treatment lowered blood glucose to levels that approached levels observed in non-diabetic (ND) mice (Fig. 1B). While insulin increased body mass, it did not restore it to the weight of ND mice (Fig. 1A).

**Table 1:** P-values from repeated measures analysis of variance (ANOVA) of weekly body mass and blood glucose between 10 weeks or 15 weeks of age and age at euthanasia

Age range:	10 weeks to 22 weeks ^a^		15 weeks to 22 weeks ^b^	
Fixed effects	Body mass	Blood glucose	Body mass	Blood glucose
Age	< 0.0001	< 0.0001	0.0152	0.0062
Canagliflozin	0.8618	< 0.0001	0.7608	0.3032
Insulin	0.0001	< 0.0001	< 0.0001	< 0.0001
Age x Cana	0.2313	< 0.0001	0.4236	0.0652
Age x Insulin	< 0.0001	< 0.0001	< 0.0001	0.0021
Cana x Insulin	0.5915	0.2377	0.556	0.1722
Age x Cana x Insulin	0.0605	0.1470	0.2454	0.3487

^a^ Canagliflozin treatment started at 9.86 weeks of age (9w 6d)

^b^ Insulin therapy started at 14.14 weeks of age (14w 1d)

Five of the untreated 14 T1D mice either died or needed to be euthanized before 22-weeks of age because they lost more than 20% of their body weight. Similarly, 4 of the 10 non-insulin treated T1D mice died or were euthanized before 22-weeks of age for the same humane end-point, despite receiving Cana for one month. All mice given insulin therapy, regardless of Cana treatment, survived until endpoint. Taking the average of the weekly measurements between 19 and 22 weeks of age (i.e., last 4 weeks of the study) for the mice that survived, only insulin significantly affected blood glucose and body mass (Table 2). Insulin treatment increased body weight, decreased blood glucose, and decreased glycated hemoglobin A1c (HbA1c), regardless of Cana pre-treatment, but it did not return the levels to that of ND mice (Table 3).

**Table 2:** P-values from two-way analysis of variance (ANOVA) of end-point measurements

Property	Canagliflozin	Group	Interaction
In vivo* measurements near euthanasia*			
Body mass (BM) ^a,b^	0.7975	** < 0.0001**	0.8412
Blood glucose (BG) ^a,b^	0.4580	** < 0.0001**	0.3605
*Serum and plasma*			
Glycated hemoglobin A1c (HbA1c)	0.6803	** < 0.0001**	0.7687
Bone resorption marker (CTX) ^b^	**0.0063**	*0.0596*	0.2032
Bone formation marker (P1NP) ^b^	**0.0013**	** < 0.0001**	**0.0022**
*Distal femur metaphysis*			
Bone volume fraction (BV/TV)	0.2329	** < 0.0001**	0.1282
Trabecular thickness (Tb.Th) ^b^	0.2673	** < 0.0001**	0.7954
Trabecular number (Tb.N) ^b^	0.9791	**0.0037**	0.6905
Trabecular spacing (Tb.Sp) ^b^	0.8393	**0.0055**	0.7421
Connectivity density (Conn.D) ^b^	0.5022	**0.0062**	**0.0172**
Trabecular tissue mineral density (Tb.TMD)	0.0884	** < 0.0001**	0.5853
*5th Lumbar vertebral body*			
Bone volume fraction (BV/TV)	0.3336	** < 0.0001**	0.8119
Trabecular thickness (Tb.Th)	0.6200	** < 0.0001**	0.8502
Trabecular number (Tb.N)	0.6344	**0.0179**	**0.0328**
Trabecular spacing (Tb.Sp)	0.4616	**0.0096**	**0.0245**
Connectivity density (Conn.D) ^b^	0.8045	0.7415	0.4155
Trabecular tissue mineral density (Tb.TMD) ^b^	0.5408	** < 0.0001**	0.7962
Cross-sectional bone area (VB.Ar)	0.3216	** < 0.0001**	0.7610
Ultimate load ^c^	0.9239	** < 0.0001**	0.5292
Estimated failure load ^d^	0.9213	** < 0.0001**	0.7061
*Femur mid-diaphysis*			
Cortical bone area (Ct.Ar) ^b^	0.7937	** < 0.0001**	0.8820
Total cross-sectional area (Tt.Ar)	0.5685	** < 0.0001**	0.7162
Minimum moment of inertia (I_min_)	0.6391	** < 0.0001**	0.8871
Section modulus (I_min_/c_min)_	0.5037	** < 0.0001**	0.8581
Cortical thickness (Ct.Th)	0.9585	** < 0.0001**	0.6646
Cortical porosity (Ct.Po) ^b^	0.1864	** < 0.0001**	0.6157
Cortical tissue mineral density (Ct.TMD) ^b^	**0.0450**	** < 0.0001**	0.6157
Stiffness ^b^	0.3186	** < 0.0001**	0.4123
Yield force	0.1170	** < 0.0001**	0.8144
Ultimate force	0.6920	** < 0.0001**	0.8070
Post-yield displacement (PYD)	0.6795	0.1196	0.6943
Work-to-fracture (W_f_)	0.8101	**0.0187**	0.6636
Post-yield W_f_	0.5991	**0.0364**	0.5919
Crack initiation toughness (K_c_)	**0.0180**	** < 0.0001**	0.5514
*Whole femur*			
Femur length ^b^	0.1872	** < 0.0001**	0.4578
Anterior–posterior width	0.0746	** < 0.0001**	0.7978
Volume fraction of bound water (BW)	0.1047	** < 0.0001**	0.6813
Fluorescent advanced glycation end-products (fAGEs)	0.1064	0.9866	0.9045
Pentosidine concentration (PEN)	0.6587	0.4337	0.9910
Pyridinoline (PYD)	0.3761	0.6501	0.9071
Deoxy-pyridinoline (DPD) ^b^	0.3704	*0.0952*	0.4267

^a^ Analyzed the average values over the last 4 weeks of life

^b^ P-values come from a non-parametric ANOVA because the residuals were not homoscedastic and/or did not come from a normal distribution. Otherwise, they come from a parametric ANOVA

^c^ Maximum force during compression testing

^d^ Estimated failure load from micro-finite element analysis

**Table 3:** Median (interquartile range) of end-point body mass and selected blood-derived measurements of metabolism

	No Canagliflozin			Adjusted *p*-values			Canagliflozin			Adjusted *p*-values		
Property (units)	ND-Palm^1^ (*n* = 10)	T1D-Palm^2^ (*n* = 8)	T1D-Ins^3^ (*n* = 10)	1 vs. 2	1 vs. 3	2 vs. 3	ND-Palm^4^ (*n* = 10)	T1D-Palm^5^ (*n* = 6)	T1D-Ins^6^ (*n* = 14)	4 vs. 5	4 vs. 6	5 vs. 6
BM (g) ^a,a^	31.6 (28.4, 33.3)	21.1 (18.7, 22.1)	25.5 (23.7, 26.3)	< 0.0001	< 0.0001	0.0002	31.2 (28.8, 34.2)	21.4 (19.8, 22.9)	25.3 (23.2, 26.4)	< 0.0001	< 0.0001	0.0157
BG (mg/dl) ^b,b^	176 (175, 181)	723 (712, 743)	280 (240, 350)	< 0.0001	0.0213	0.0610	174(170, 183)	727 (674, 740)	324 (228, 408)	< 0.0001	0.0298	0.0191
HbA1c (%) ^a,a^	4.04 (3.40, 4.44)	9.93 (9.36, 10.3)	5.27 (5.06, 5.90)	< 0.0001	< 0.0001	< 0.0001	3.84 (3.58, 4.10)	9.80 (9.43, 10.2)	5.50 (4.98, 6.22)	< 0.0001	< 0.0001	< 0.0001
CTX (ng/ml) ^b,b^	10.3 (9.5, 13.3)	13.8 (10.2, 75.2)	16.2 (13.7, 31.6)	0.3467	0.0368	> 0.99	9.87 (9.53, 11.4)	12.8 (10.2, 15.6)	10.7 (9.31, 13.7)	0.2627	> 0.99	0.5954
P1NP (ng/ml) ^b,b^	15.4 (13.0, 23.6)	3.31 (2.78, 8.42)	39.1 (28.1, 52.7)	0.0349	*0.0618*	< 0.0001	15.8 (12.7, 20.3)	4.51 (3.62, 4.93)	18.5 (14.0, 30.5)	0.0096	> 0.99	0.0005

^a,a^ Holm-Šídák's multiple comparisons test of the 3 groups within No Cana or Cana (2 families)

^b,b^ Dunn's multiple comparisons test of the 3 groups within No Cana or Cana (2 families)

Body mass (BM); Blood glucose (BG); glycated hemoglobin A1c (HbA1c); C-terminal telopeptide crosslink of collagen I (CTX); and Procollagen type I N-terminal propeptide (P1NP)

### Pre-treating Mice with Canagliflozin (Cana) Aided Insulin’s Ability to Improve Bone Metabolism in T1D Mice

With regards to serum markers of bone metabolism, Cana pre-treatment lowered bone resorption (CTX), but this serum marker did not strictly depend on the 3 glucose groups (*p* = 0.0596). Nonetheless, in the non-parametric post-hoc, pairwise comparisons (Dunn’s test), CTX was unexpectedly higher in T1D-Ins than in ND-Palm but not significantly different between T1D-Ins and T1D-Palm without Cana in the chow (Table 3). There were no pair-wise differences in CTX among these glucose groups when Cana was in the chow for a month (Table 3) suggesting the glucose-lowering effect of this drug normalized bone resorption in T1D. Cana pre-treatment also significantly affected bone formation (P1NP), and the insulin effect on P1NP depended on the Cana pre-treatment (interaction *p*-value = 0.0022; Table 2). Compared to non-diabetic mice without Cana, T1D significantly decreased P1NP while insulin therapy increased P1NP (Table 3). Cana pre-treatment normalized P1NP levels between ND-Palm and T1D-Ins groups similar to its effect on CTX (Table 3).

### Insulin Therapy Partially Rescued the T1d-Related Loss in Trabecular Bone Volume and Architecture, and Pre-treatment with Cana Helped Insulin to Improve Trabecular Number

**Fig. 2:**
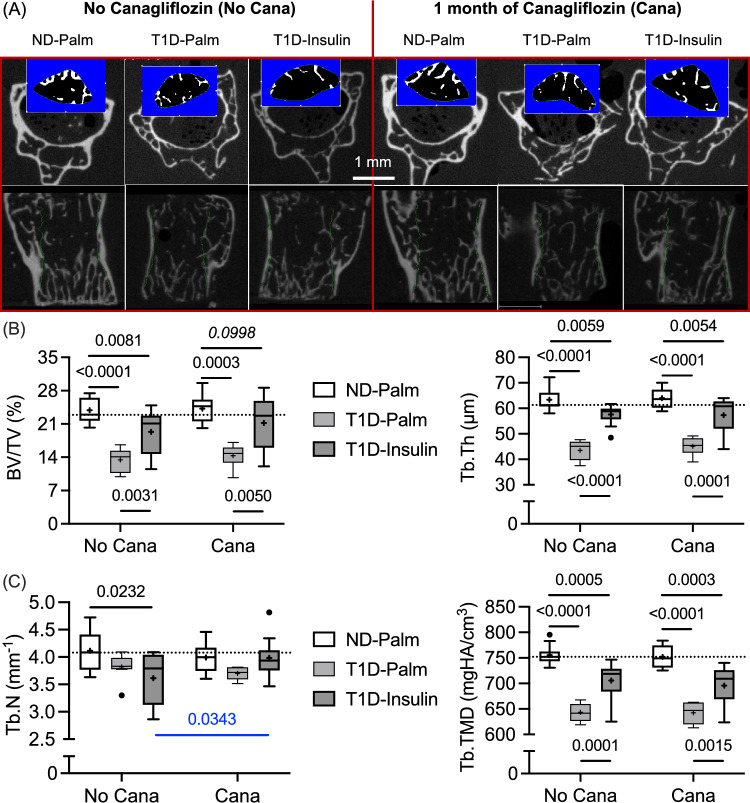
Differences in micro-computed tomography parameters of the L5 vertebral body. **A** As defined by the contours (green), the trabecular bone within each vertebral body was evaluated. **B** The amount of trabecular bone was significantly lower in T1D mice, not treated with insulin (Palm) because the reduction in insulin production reduced the thickness of the trabeculae. **C** Mice pre-treated with Cana before insulin therapy had more trabeculae (Tb.N) than mice only receiving insulin therapy. Regardless of Cana pre-treatment, insulin therapy partially rescued the T1D-related decrease in tissue mineral density. The horizontal dashed line is the median of non-diabetic mice without Cana pre-treatment

As expected, the STZ-induced T1D decreased trabecular bone volume fraction (BV/TV) and trabecular thickness (Tb.Th) in both the distal femur metaphysis (Table S2) and the centrum of the 5th lumbar (L5) vertebral body (Fig. 2A). Insulin therapy partially rescued this loss, but BV/TV and Tb.Th were still significantly lower in T1D-Insulin group than in the ND-Palm group, regardless of Cana (Fig. 2B). Interestingly, trabecular number (Tb.N) of the L5 VB was significantly lower in the T1D-Ins group than in the ND-Palm group when mice did not receive Cana before the start of insulin therapy (Fig. 2C). This was not the case for Cana-treated mice such that Tb.N was the same between ND-Palm and T1D-Ins and was higher in T1D-Ins with Cana pre-treatment than in T1D-Ins without Cana pre-treatment (Fig. 2C). Regardless of anatomical site, the Cana pre-treatment did not worsen the T1D-related decline in trabecular tissue mineral density (Tb.TMD), and insulin partially rescued the T1D-related decline in Tb.TMD (Table S2 and Fig. 2C). Connectivity density (Conn.D) in the distal femur metaphysis was the only architectural parameter for which the interaction between glucose group and Cana was significant (*p* = 0.0172 in Table 2). It was significantly lower in T1D-Palm than in ND-Palm without Cana pre-treatment, but this was not the case in mice with Cana pre-treatment (Table S2).

### Cana Pre-treatment Had no Effect on Cortical Bone Structure nor the Ability of Insulin Therapy to Reverse T1D-Related Deficits in Cortical Bone Structure

**Fig. 3:**
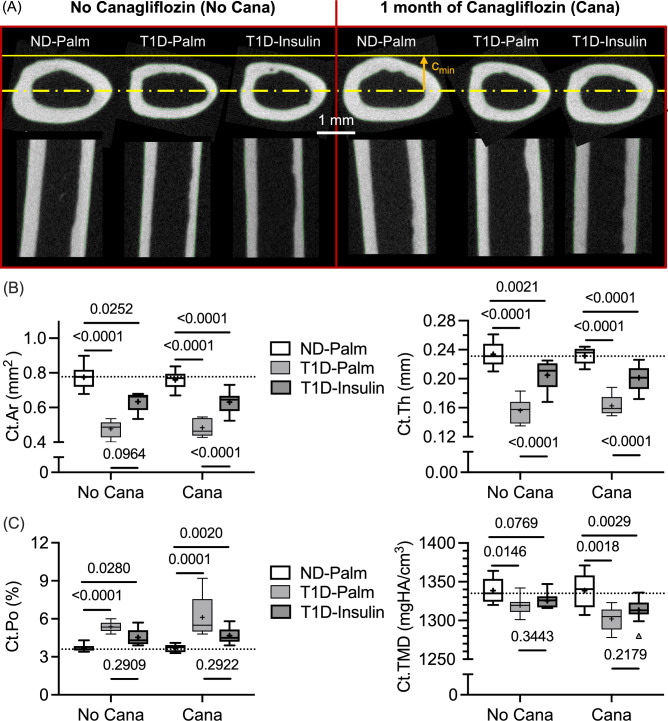
Differences in micro-computed tomography parameters of the femur mid-diaphysis. **A** As defined by the contours (endosteal and periosteal surfaces), the cortical bone of the diaphysis was evaluated. **B** The cross-sectional area of bone (Ct.Ar) and the cortical thickness (Ct.Th) were significantly lower in T1D mice, not treated with insulin. Insulin therapy partially rescued this decline. **C** T1D also increased cortical porosity (Ct.Po) while it decreased cortical tissue mineral density (Ct.TMD). Insulin therapy did not reverse these T1D-related changes, regardless of Cana pre-treatment. The horizontal dashed line is the median of non-diabetic mice without Cana pre-treatment

With respect to the cortical bone of the femur mid-diaphysis (Fig. 3A), Cana pre-treatment did not affect any structural parameters (Table 2). Insulin therapy partially rescued cortical bone area (Ct.Ar) and cortical thickness (Ct.Th) as well as femur length and the anterior–posterior width of the mid-diaphysis (Table S2). Insulin however had minimal effect on cortical porosity (Ct.Po) and Ct.TMD (Fig. 3C). Although Cana pre-treatment appeared to lower this μCT measurement of tissue-level mineral density (Cana *p*-value = 0.0251 in Table 2), none of the post hoc pairwise comparisons (No Cana vs. Cana within each glucose group) were significant (adjusted-*p*-value > 0.142).

### Insulin Therapy, Not Cana Pre-Treatment, Increased the Fracture Resistance of Diabetic Bone

**Fig. 4:**
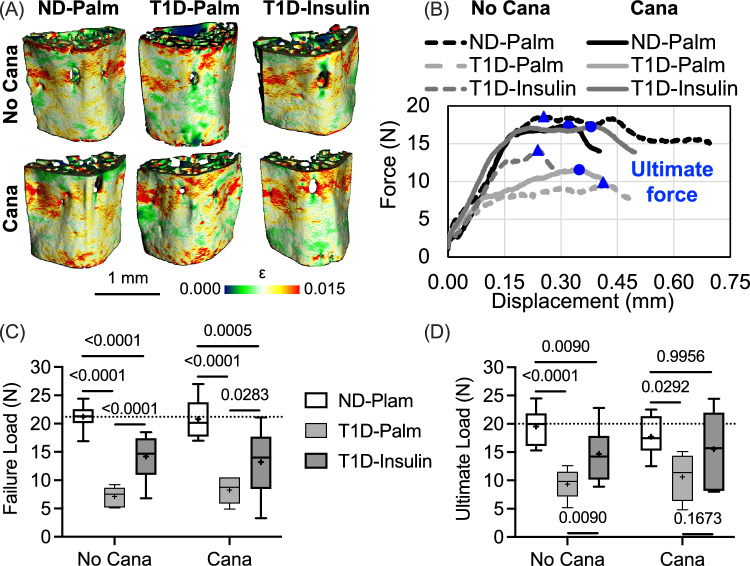
Differences in compressive mechanical properties of lumbar vertebral body (VB). **A** From the distribution of equivalent strain in the finite element models of VBs subjected to an apparent strain of 1% in compression, the failure load was estimated as the reaction force that causes 2% of the bone voxels (elements) to exceed 0.007 strain (ε). **B** VB strength was determined from force vs. displacement curves as the ultimate load (blue symbols) that the bone endures during compressive loading. **C**,**D** These measurements of strength were significantly lower in T1D mice. Insulin therapy partially rescued this decline. The horizontal dashed line is the median of non-diabetic mice without Cana pre-treatment. The dashed lines on the force vs. displacement curves are for no Cana pre-treatment

Consistent with the treatment effects on trabecular bone, Cana pre-treatment did not affect the compressive strength of the L5 vertebral body (Table 2), as estimated by micro-finite element analysis (Fig. 4A) and compression testing (Fig. 4B). Insulin therapy partially rescued the estimated failure load of the L5 VB (Fig. 4C), irrespective of Cana pre-treatment. Ultimate load was significantly higher in the T1D-Ins group than in the T1D-Palm group when the mice did not receive Cana (Fig. 4D). This same insulin-related difference was not observed in the Cana pre-treated mice because there was more variability (i.e., higher interquartile range) in ultimate force such that it did not significantly vary between ND-Palm and T1D-Ins groups (Fig. 4D).

**Fig. 5:**
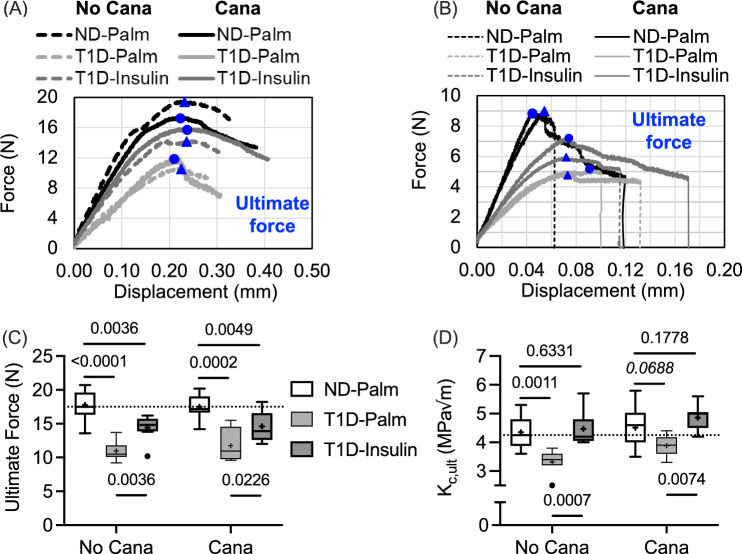
Differences in the flexural mechanical properties of the femur mid-diaphysis. **A** In representative force vs. displacement curves from three-point bending tests, T1D bones are weaker than non-diabetic bones as indicated by the ultimate force (blue symbols). **B** In representative force vs. displacement curves from crack propagation tests (see Fig. S2), the notched femur sustained less force (blue symbols) when from T1D mice, irrespective of canagliflozin treatment. **C**.**D** T1D reduced strength (bottom left) and fracture toughness (bottom right), while insulin therapy partially rescued these mechanical properties. The horizontal dashed line is the median of non-diabetic mice without Cana pre-treatment. The dashed lines on the force vs. displacement curves are for no Cana pre-treatment

With respect to cortical bone of the femur mid-diaphysis, ultimate force during three-point bending (Fig. 5A) was significantly lower in T1D-Palm mice compared to ND-Palm mice (Table 2 and Fig. 5C). This was the case even when mice received a month of Cana pre-treatment. Glucose group but not Cana affected stiffness, yield force, work-to-fracture (W_f_), and post-yield (PY) W_f_, while neither Cana nor glucose group affected post-yield displacement (Table 4). Like ultimate force, T1D decreased stiffness, yield force, and W_f_ with insulin therapy partially rescuing these deficits (Table 4). Interestingly, the ability of cortical bone to resist crack growth (crack initiation toughness or K_c,ult_) during three-point bending (Fig. 5B) depended on Cana pre-treatment and insulin therapy, but not their interaction (Table 2). However, in the post-hoc comparisons between No Cana and Cana, K_c,ult_ was not significantly higher (adj-*p*-value > 0.172) when mice received Cana pre-treatment (Fig. 5D).

**Table 4:** Median (interquartile range) of bone properties from three-point bending of the femur diaphysis

	No Canagliflozin			Adjusted *p*-values			Canagliflozin			Adjusted *p*-values		
Property (units)	ND-Palm^1^ (*n* = 9)	T1D-Palm^2^ (*n* = 7)	T1D-Ins^3^ (*n* = 8)	1 vs. 2	1 vs. 3	2 vs. 3	ND-Palm^4^ (*n* = 10)	T1D-Palm^5^ (*n* = 4)	T1D-Ins^6^ (*n* = 13)	4 vs. 5	4 vs. 6	5 vs. 6
Stiffness (N/mm) ^b,b^	112 (101, 128)	60 (56, 73)	87 (82, 94)	0.0002	0.1361	0.1169	116 (108, 125)	51 (72, 91)	84 (81, 93)	0.0023	0.0020	0.9767
Yield force (N) ^a,a^	14.2 (13.2, 15.7)	9.90 (8.40, 11.0)	13.4 (10.8, 14.7)	0.0003	0.0626	0.0223	16.0 (15.1, 17.1)	10.2 (8.8, 13.0)	13.2 (11.9, 14.9)	0.0003	0.0040	0.0304
PYD (mm) ^c^	0.193 (0.109, 0.256)	0.087 (0.073, 0.453)	0.291 (0.200, 0.402)	N/A	N/A	N/A	0.128 (0.051, 0.189)	0.228 (0.068, 0.392)	0.262 (0.119, 0.344)	N/A	N/A	N/A
W_f_ (mJ) ^a,a^	4.74 (2.96, 5.64)	1.89 (1.41, 3.92)	4.29 (3.66, 6.11)	0.0401	0.6510	0.0269	3.66 (2.39, 4.51)	3.34 (1.62, 3.95)	4.88 (3.05, 5.22)	0.5853	0.5853	0.3992
PY W_f_ (mJ) ^b,a^	3.51 (1.82, 4.64)	0.87 (0.78, 2.83)	3.42 (2.58, 4.77)	0.1378	> 0.99	0.0605	2.14 (0.85, 3.23)	2.18 (0.84, 2.82)	3.19 (1.80, 4.18)	0.0691	0.5298	0.5298

^a,a^ Holm-Šídák's multiple comparisons test of the 3 groups within No Cana or Cana (2 families)

^b,b^ Dunn's multiple comparisons test of the 3 groups within No Cana or Cana (2 families)

^b,a^ Dunn's multiple comparisons test of the 3 groups within Cana (1 family) and Holm-Šídák's multiple comparisons test of the 3 groups within No Cana (1 family)

^c^ Not applicable (N/A) because the two-way ANOVA indicated that Cana pre-treatment nor group significantly affect the property

Post-yield displacement (PYD); Work-to-fracture (W_f_); and post-yield work-to-fracture (PY W_f_)

### T1D Decreased Bound Water, But it Did Not Increase Advanced Glycation End-Products in the Bone Matrix

**Fig. 6:**
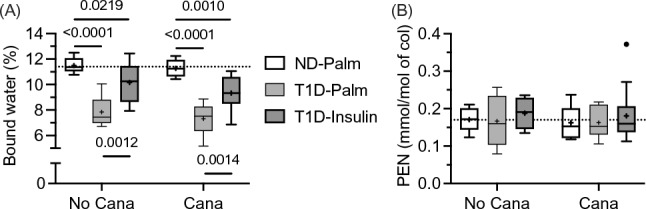
Differences in matrix characteristics of cortical bone. **A** The volume of bound water bound to the matrix was quantified from NMR-derived T_2_ spectra (see Fig. S1). T1D decreased volume of bound water per bone volume, irrespective of Cana pre-treatment; and insulin therapy partially rescued this decline. **B** Mature collagen crosslinks within the organic matrix were quantified from HPLC-derived chromatograms (see Fig. 3S). T1D, Cana pre-treatment, nor insulin therapy affected pentosidine concentration or the level of this glycation-mediated, non-enzymatic collagen crosslink. The horizontal dashed line is the median of non-diabetic mice without Cana pre-treatment

Beyond diabetes affecting the cortical structure and trabecular architecture of bone, T1D could alter characteristics of the bone matrix or bone tissue, especially since circulating glucose was high in the T1D mice compared to the ND mice (Fig. 1B and Table 3). Despite this, neither T1D, Cana pre-treatment, nor insulin therapy affected fluorescent advanced glycation end-products (Table 2 and Table S2) and pentosidine levels (Table 2 and Fig. 6B) in the organic matrix of bone. Likewise, there were no significant differences in the mature enzymatic collagen crosslinks among the Cana and glucose groups (Table 2 and Table S2). The level of bone matrix hydration or bound water, on the other hand, was significantly lower in the T1D mice than in the ND mice, regardless of Cana pretreatment (Table 2 and Fig. 6). Insulin therapy partially rescued this T1D-related decrease in bound water with Cana pre-treatment having no effect on this matrix characteristic (Fig. 6A).

## Discussion

Refuting our supposition, reducing the levels of circulating glucose did not affect the ability of insulin therapy to rescue the T1D-related decrease in bone strength. Conceivably, the degree and severity of hyperglycemia in T1D could mediate metabolic changes (e.g., oxidative damage to mitochondria) that might lessen the ability of cells to respond to exogenous insulin [23, 40]. In mice, this does not appear to be the case for bone strength, at least not for a duration of 5-to-6 weeks of hyperglycemia before the start of insulin therapy (Fig. 1B). Lowering of blood glucose by an SLGT2 inhibitor prior to insulin therapy was, nonetheless, beneficial for the serum marker of bone resorption (Table 3) and the crack initiation toughness of cortical bone (Fig. 5D). Cana pre-treatment helped insulin normalize the T1D-related perturbations in bone turnover markers (Table 3) and protected the number of trabeculae in the lumbar vertebra (Fig. 2C).

In our previous study involving the STZ model of T1D in male DBA/2J mice, continuous infusion of insulin using osmotic minipumps prevented the T1D decline in bone strength in a dose-dependent manner [13]. In the same study, insulin therapy at 0.25 IU/day after a 4-week delay rescued the ultimate force endured by the femur during three-point bending [13]. Treating STZ-induced T1D with both insulin at 0.125 IU/day and Cana in chow at 50 ppm for 8 weeks increased bending strength of the femur mid-diaphysis (i.e., ultimate force), but this did not return strength to the level of age-matched, non-diabetic male mice, despite the combination therapy having the best glucose control [14]. Insulin therapy using subcutaneous implants with or without Cana pre-treatment in the present study also did not fully rescue this measurement of cortical bone strength (Fig. 4D). Lowering systemic glucose with insulin helps reduce mortality of animals following STZ injections [41], but consistent dosing (e.g., 0.25 IU/day) is likely important for exogenous insulin to be the most anabolic and promote optimal bone formation and strength in T1D.

Canagliflozin is an approved therapy for glycemic control in those with type 2 diabetes and effectively lowers glucose levels and minimizes cardiovascular complications [42]. Because the drug blocks the reabsorption of glucose and sodium in the kidney, calcium excretion and phosphate reabsorption accompanies the glucosuria that SGLT2 inhibitors cause [14]. This imbalance in circulating electrolytes could increase systemic parathyroid hormone (PTH) and subsequent bone resorption. Treating non-diabetic, male mice with Cana for 10 weeks however did not increase serum parathyroid hormone and CTX (bone resorption marker) nor decrease tissue mineral density of cortical and trabecular bone [19]. Inactivating the function of SGLT2 in mice at conception drastically increased calcium excretion and decreased TMD of cortical and trabecular bone, but this did not lead to weaker bone [43]. Treating genetically diverse mice (UM-HET3) with Cana long-term from 7 to 22 months age decreased the degree of mineralization of cortical bone, namely in male mice [44]; whereas treating UM-HET3 mice at 6 months of age for 1-, 3,-, or 6-months did not affect serum PTH, nor cortical TMD and thickness in male and female mice [45]. In young and adult C57BL/6 J mice, Cana treatment for 6 months also did not affect serum PTH and had positive effects on bone morphology, despite an increased urinary calcium excretion [46]. The present study supports the benign effect of short-term Cana-related glucosuria on bone resorption. In the current study, 4 weeks of Cana before insulin therapy significantly decreased CTX while its effect on P1NP depended on glucose group (Table 2). Regardless, it brought these serum markers of bone turnover in the insulin-treated mice closer to the levels observed in the non-diabetic mice. There may be other positive effects of Cana treatment, beyond lowering circulating glucose, that promote strong bone.

The accumulation of advanced glycation end-products (AGEs) is postulated to contribute to bone fragility in diabetes because the irreversible formation of glycation-mediated, non-enzymatic collagen crosslinks conceivably reduces toughening mechanisms inherent in healthy bone [47]. In the only study to biochemically measure collagen crosslinks in the bones of mice, PEN was significantly higher while post-yield work-to-fracture (four-point bending) was significantly lower in STZ-induced T1D mice than in control mice [48]. This occurred when the mice were euthanized after 7 weeks of hyperglycemia, but not after 3 weeks of hyperglycemia. Despite similar blood glucose and HbA1c levels between the present study and the previous study [48], we did not detect differences in PEN and found post-yield work-to-fracture to be marginally lower in T1D than in ND (adjusted-*p* value = 0.1378) without Cana (Table 4). Several factors could explain the discrepancies between the 2 studies (e.g., differences in inbred strain, STZ dose, age at first STZ injection, and HPLC assay). Most notably in the previous study, PEN varied from 0.0003 to 0.003 mmol/mol of collagen in the control group and varied from 0.003 to 0.005 mmol/mol in the T1D group. These levels are quite low. In contrast, PEN varied between 1.02 to 1.64 mmol/mol in human control bone from elderly donors and between 1.41 to 2.09 mmol/mol in cortical bone from age-matched donors with T1D, for which post-yield toughness was lower compared to the sex- and age-matched control donors [49]. Unlike human studies, the duration of hyperglycemia in mouse models may not be long enough to cause a meaningful accumulation of AGEs to explain any decrease in bone toughness.

Although we did not observe any T1D-related differences in AGEs, we did find that T1D decreased both bound water and crack initiation toughness (K_c,ult_), two matrix-sensitive characteristics of bone (i.e., independent of bone structure unlike ultimate force). To the best of our knowledge, only one other study assessed the effect of T1D on the fracture toughness of cortical bone and found that the ability of bone to resist crack growth (K_c,ult_) was lower in female transgenic OVE26 mice that develop severe T1D at birth than in non-diabetic female FVB mice [50]. They attributed this difference in fracture toughness to Raman spectroscopy (RS)-derived measures of mineral-to-matrix ratio (lower v_2_PO_4_/amide III in T1D mice), CML (higher integrated area at 1150 cm^−1^ per integrated area at 1450 cm^−1^ in T1D mice), and PEN (higher integrated area at 1495 cm^−1^ per integrated area at 1450 cm^−1^ in T1D mice). These AGE bands are, however, weak in RS and not readily detectable [51], thereby requiring a highly sensitive RS instrument. In the present study, the lower K_c,ult_, which was rescued by insulin treatment (Fig. 4D), was likely related to the T1D-related decrease in bound water volume per bone volume, which was partially rescued by insulin therapy (Fig. 5B). Notably, Cana significantly increased K_c_ (Fig. 5D) but did not affect BW (Fig. 6A) when controlling for glucose group in the two-way ANOVA (Table 2).

The effect of T1D on the level of matrix hydration in bone is unknown. We speculate that the STZ-related decline in insulin decreases the amount of glycosaminoglycans (GAGs) since GAGs can hold water via hydrogen bonding. As assessed by RS, GAG levels were lower in cadaveric bone from donors with long-term T1D than from donors without diabetes [49]. In genetic mice lacking 2 proteoglycans that bind GAGs, decorin and/or biglycan, the GAG content, bound water level, and crack initiation toughness of long bones, were all lower in single and double knock-out animals compared to wild-type animals [52]. In the present study, insulin treatment over 8 weeks partially rescued BW suggesting that the matrix is modifiable in T1D. We speculate that osteocytes can respond to exogeneous insulin and confer modifications to the matrix that promotes bound water. This is based on observations from mouse models of T1D. For example, lacunar density was lower in male Akita mice (spontaneously develop T1D at 5 weeks of age) than in age- and sex-matched, 20-week-old C57BL/6J mice [53]. In the T1D mouse study that is similar to the present study, but using a different inbred strain, the number of canalicular branches (i.e., multiple channels branching off the primary canalicular channel) and the number of canalicular intersections (i.e., nodes) was lower in the femur diaphysis from T1D mice than from non-diabetic control mice [48]. In this study, lacunar density non-significantly trended toward being lower in T1D group than in the control group and negatively correlated with HbA1c (*r* = −0.39, *p* < 0.05). Whether by a T1D-related decrease in vascular channel volume or decrease in the number of lacunae connected to each other with multiple branching canaliculi, the decline in insulin could potentially decrease the delivery of a matrix factors like GAGs to the matrix.

In addition to not assessing the lacunar-canalicular network, GAG content, and vascularity, another limitation of the present study was the lack of assessments related to other important mediators of glucose and bone metabolism that contribute to diabetic bone disease. In animal models of T1D and in humans with T1D, sclerostin and inflammatory cytokines appear to be elevated (reviewed in [54]). Whether hyperglycemia or low insulin primarily facilitates the inhibition of bone formation and promotion of bone resorption in T1D remains to be elucidated. Exogenous insulin, endogenous insulin, and endogenous IGF-1 were also not measured, so we cannot determine if systemic insulin and IGF-1 levels, with or without Cana pre-treatment, is directly proportional to bone strength. Like insulin, IGF-1 is lower in T1D [55] and conventional insulin therapy does not restore IGF-1 to normal levels like intraportal insulin infusion (i.e., the liver primarily generates IGF-1 [55, 56]); and exogenous IGF-1 treatment improved bone strength in T1D mice [57].

There are several other aspects of the present study that influence interpretation of the reported findings. Without measuring blood ketone levels, the present study cannot exclude ketosis as mediator of bone loss, an observation in rodents fed a ketogenic diet [58, 59]. There is a possibility that our rehydration protocol did not fully remove the ethanol, a solvent that decreases post-yield toughness but increases crack initiation toughness [31, 32], but presumably, Cana pre-treatment, T1D, and insulin therapy did not affect how the matrix rehydrates as the concentration of ethanol decreased to 100% water. Reassuringly, the trends in bound water (Fig. 6A) matched the trends in crack initiation toughness (Fig. 5D), albeit insulin treatment fully restored K_c,ult_ to ND levels while bound water remained significantly lower in T1D-Insulin than in ND-Palm. Lastly, the present findings cannot be extended to female mice or other strains of mice.

## Conclusion

Lowering glucose levels by canagliflozin, an SLGT2 inhibitor that causes the excretion of glucose and lowers blood glucose, for 4 weeks prior to the start of insulin therapy did not improve the ability of subcutaneous insulin to rescue the T1D-related decline in bone strength that occurs in a mouse model of this disease. While Cana pre-treatment can have a modest negative effect on TMD of cortical bone (significant when pooling diabetic groups), it normalizes serum markers of bone turnover and favors higher crack initiation toughness. Lastly, 13-to-14 weeks of hyperglycemia did not increase advanced glycation end-products in the femur of T1D mice, but it did significantly lower matrix-bound water, which was partially recovered with insulin therapy. Overall, glucose levels prior to insulin treatment does not appear to affect the ability of exogenous insulin to prevent bone fragility in T1D mice.

## Supplementary Information

Below is the link to the electronic supplementary material.

Supplementary file1 (DOCX 1197 KB)

## Data Availability

Data herein is available upon request.
