# Supplementary material for: Effect of Canagliflozin Pretreatment on the Efficacy of Insulin Therapy to Rescue Type 1 Diabetes-Related Bone Fragility in Male Mice
Source: Calcif Tissue Int. 2026 Jan 31;117(1):20. doi: 10.1007/s00223-026-01486-x (PMC12858567; doi:10.1007/s00223-026-01486-x)
Supplement: Supplementary file 1 — Supplementary file1 (DOCX 1197 KB) [file 223_2026_1486_MOESM1_ESM.docx]

**The Effect of Prior Glycemia on the Efficacy of Insulin Therapy to Rescue Type 1 Diabetes-Related Bone Fragility in Mice**

Jeffry S. Nyman, R. Clay Bunn, Sasidhar Uppuganti, Elizabeth M. Hennen, Philip D. Ray, Anthony Garcia Mendez, Evangelia Kalaitzoglou, and John L. Fowlkes

**Table S1**. Scan and segmentation parameters for ex vivo μCT imaging of mouse bones

| Parameter | Units | Femur mid-diaphysis | Distal femur metaphysis | Notched femur mid-diaphysis | L6 Vertebra |
| --- | --- | --- | --- | --- | --- |
| Peak voltage of X-ray tube | kVp | 70 | 70 | 70 | 70 |
| Current of X-ray tube | μA | 200 | 114 | 114 | 200 |
| Integration time | s | 0.3 | 0.3 | 0.6 | 1.2 |
| Samples per projection | *Pixels* ^a^ | 1024 | 1024 | 1024 | 1024 |
| Projections | *No.* ^a^ | 1000 | 1000 | 1000 | 1000 |
| Al filter thickness | mm | 0.5 | 0.5 | 0.5 | 0.5 |
| Voxel size | μm | 6 | 6 | 6 | 10 |
| Long-axis (z) dimension | *Slices* ^a^ | 310 | 450 | 310 | 137-247 |
| Gaussian sigma | - | 0.8 | 1.0 | 0.2 | 1.0 |
| Gaussian support | *-* ^a^ | 2 | 2 | 1 | 2 |
| Lower threshold | mg·HA/cm^3^ | 1149.6 | 390.3 | 837.0 | 315.9 |
| Upper threshold | mg·HA/cm^3^ | 2787.3 | 2787.3 | 2787.3 | 2787.3 |

^a^ Integer value

**Table S2**. Median (interquartile range) of bone properties from micro-computed tomography evaluations.

|  | **No Canagliflozin** | | | Adjusted p-values | | | **Canagliflozin** | | | Adjusted p-values | | |
| --- | --- | --- | --- | --- | --- | --- | --- | --- | --- | --- | --- | --- |
| Property (units) | ND-Palm^1^  (n=10) | T1D-Palm^2^  (n=8) | T1D-Ins^3^  (n=9) | 1 vs. 2 | 1 vs. 3 | 2 vs. 3 | ND-Palm^4^ (n=10) | T1D-Palm^5^  (n=5-6) | T1D-Ins^6^  (n=14) | 4 vs. 5 | 4 vs. 6 | 4 vs. 6 |
|  | *Whole femur* | | | | | | | | | | | |
| Length (mm)^b,b^ | 14.5 (14.2, 14.8) | 13.4 (12.6, 14.1) | 14.3 (14.1, 14.4) | 0.0002 | 0.2261 | 0.0738 | 14.7 (14.3, 14.8) | 13.7 (13.6, 13.9) | 14.3 (13.9, 14.5) | 0.0010 | 0.0394 | 0.2185 |
| A-P Width (mm)^a,a^ | 1.03 (0.97,1.09) | 0.91 (0.86, 0.93) | 0.95 (0.95,1.00) | 0.0001 | 0.0251 | 0.0251 | 1.08 (1.04, 1.11) | 0.92 (0.89, 0.95) | 1.00 (0.97, 1.02) | <0.0001 | <0.0001 | 0.0003 |
| fAGE (mgQ/mmol)^c^ | 61.4 (54.1, 76.0) | 65.3 (46.4, 75.3) | 61.9 (50.9, 69.8) | N/A | N/A | N/A | 51.2 (43.2, 67.2) | 53.6 (41.2, 74.1) | 53.6 (43.9, 62.6) | N/A | N/A | N/A |
| PYD (mmol/mol)^c^ | 389 (332, 461) | 374 (337, 452) | 420 (344, 475) | N/A | N/A | N/A | 366 (286, 487) | 352 (286, 468) | 385 (329, 423) | N/A | N/A | N/A |
| DPD (mmol/mol)^c^ | 16.7 (11.6, 22.1) | 16.2 (12.6, 24.1) | 21.6 (18.1, 24.1) | N/A | N/A | N/A | 13.4 (9.20, 19.0) | 18.9 (13.9, 28.4) | 17.1 (12.7, 21.2) | N/A | N/A | N/A |
|  | *Distal femur metaphysis* | | | | | | | | | | | |
| BV/TV (%)^a,a^ | 9.95 (8.03, 11.0 | 2.90 (2.38, 3.73) | 6.90 (5.15, 10.5) | <0.0001 | 0.0081 | 0.0031 | 7.35 (5.90, 9.65) | 3.20 (2.55, 3.95) | 7.45 (6.28, 8.68) | 0.0003 | 0.0998 | 0.0050 |
| Tb.Th (μm)^a,b^ | 58.6 (54.4, 62.8) | 35.7 (32.1, 36.8) | 48.4 (46.4, 51.0) | <0.0001 | <0.0001 | <0.0001 | 57.2 (51.6, 62.1) | 35.0 (33.1, 36.6 | 47.2 (45.4, 48.9) | <0.0001 | 0.0099 | 0.0748 |
| Tb.N (1/mm)^b,b^ | 3.03 (2.75, 3.15) | 2.56 (2.32, 2.71) | 2.77 (2.39, 3.44) | 0.0310 | >0.99 | 0.2710 | 2.81 (2.69, 3.14) | 2.55 (2.51, 2.59) | 2.98 (2.48, 3.31) | 0.2587 | >0.99 | 0.1593 |
| Tb.Sp (μm)^b,b^ | 329 (315, 361) | 390 (367, 432) | 363 (285, 432) | 0.0311 | >0.99 | 0.2716 | 355 (316, 376) | 394 (386, 398) | 334 (298, 405) | 0.2254 | >0.99 | 0.1600 |
| Conn.D (1/mm^3^)^b,b^ | 90 (56, 118) | 33 (25, 41 | 58 (35, 101) | 0.0062 | 0.6827 | 0.1870 | 42 (34, 55) | 53 (24, 61) | 80 (47, 94) | >0.99 | 0.0581 | 0.2672 |
| Tb.TMD  (mg·HA/cm^3^)^a,b^ | 721 (698, 743) | 600 (582, 616) | 654 (628, 683) | <0.0001 | <0.0001 | 0.0006 | 720 (684, 746) | 580 (563, 594) | 646 (635, 657) | <0.0001 | 0.0016 | 0.1702 |
|  | *L5 vertebral body* | | | | | | | | | | | |
| Tb.Sp (μm)^a,a^ | 231 (218, 254) | 258 (244, 263 | 257 (238, 314) | 0.1693 | 0.0201 | 0.2947 | 237 (232, 262) | 264 (258, 272) | 243 (233, 260) | 0.0919 | 0.8878 | 0.0919 |
| Conn.D  (1/mm^3^)^c^ | 127 (101, 149) | 128 (113, 139) | 100 (110, 143) | N/A | N/A | N/A | 126 (118, 138) | 121 (108, 129) | 128 (118, 136) | N/A | N/A | N/A |
|  | *Femur mid-diaphysis* | | | | | | | | | | | |
| Tt.Ar (mm^2^)^a,a^ | 1.29 (1.19, 1.36) | 1.02 (0.94, 1.03) | 1.09 (1.07, 1.11) | <0.0001 | <0.0001 | 0.0345 | 1.28 (1.19, 1.34) | 0.979 (0.89, 1.06) | 1.09 (1.04, 1.14) | <0.0001 | 0.0007 | 0.0097 |
| I_min_ mm^4^)^a,a^ | 0.073 (0.064, 0.079) | 0.040 (0.034, 0.046) | 0.055 (0.051, 0.058) | <0.0001 | 0.0001 | 0.0024 | 0.072 (0.064, 0.078) | 0.038 (0.033, 0.046) | 0.052 (0.049, 0.059) | <0.0001 | 0.0001 | 0.0016 |
| SM (mm^3^)^a,a^ | 0.137 (0.126, 0.149) | 0.087 (0.075, 0.094) | 0.113 (0.101, 0.117) | <0.0001 | 0.0002 | 0.0005 | 0.136 (0.125, 0.144) | 0.081 (0.069, 0.096) | 0.108 (0.102, 0.121) | <0.0001 | 0.0004 | 0.0004 |

^a,a^ Holm-Šídák's multiple comparisons test of the 3 groups within No Cana or Cana (2 families)

^a,b^ Holm-Šídák's multiple comparisons test of the 3 groups within No Cana (1 family) and Dunn's multiple comparisons test of the 3 groups within Cana (1 family)

^b,b^ Dunn's multiple comparisons test of the 3 groups within No Cana or Cana (2 families)

^c^ Not applicable (N/A) because the two-way ANOVA indicated that Cana pre-treatment nor group significantly affect the property

Solid

protons

Bound

water

Short T_2_ Pore water

Long T_2_ Pore water

+ Lipid

Reference water

volume

**Figure S1. Examples of the ^1^H NMR T_2_ spectra from mouse bones**. During NMR relaxometry, solid protons (^1^H) relax faster than water protons bound to the matrix, which in turn relax faster than water protons residing in the pore spaces of bone. Since the marrow was not flushed from femurs, there was a strong overlapping NMR signals from lipids. The integrated area of the bound water peak was divided by the integrated area of the reference water peak (= 20.2 μl) to obtain the volume of bound water.


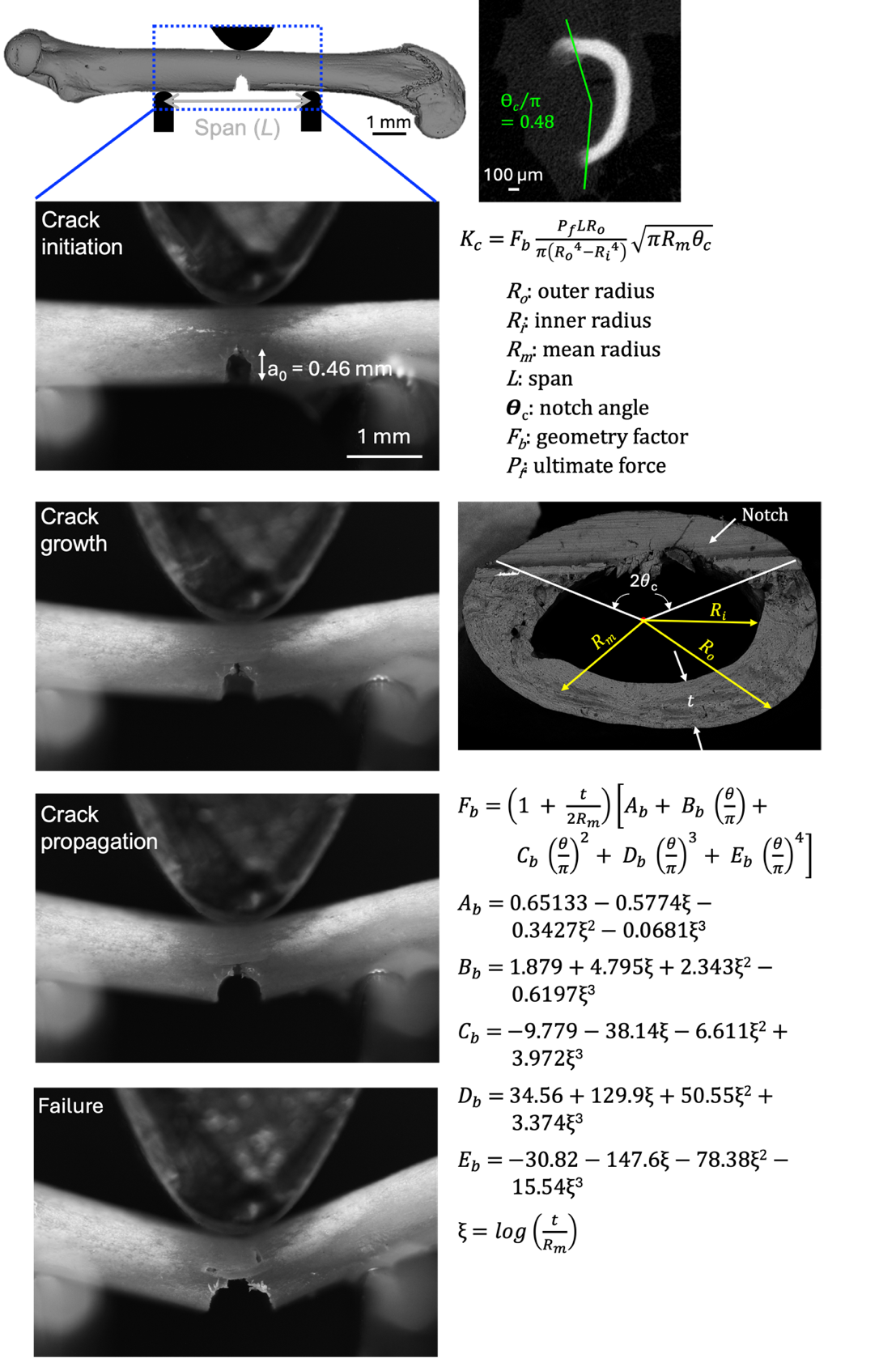


**Figure S2. Fracture toughness testing of mouse femur mid-diaphysis**. After a micro-notch is generated at the mid-point on the anterior side, it scanned by micro-computed tomography so that the notch angle can be determined. As the femur mid-diaphysis is loaded in three-point bending, a crack propagates from the micro-notch to the posterior side. The equation to determine crack imitation toughness comes from fracture toughness testing of thinned-walled metal cylinders. The labeling of geometrical parameters on the image from scanning electron microscopy is an illustration. These are determined from the μCT images of the notched region.

Reference water volume


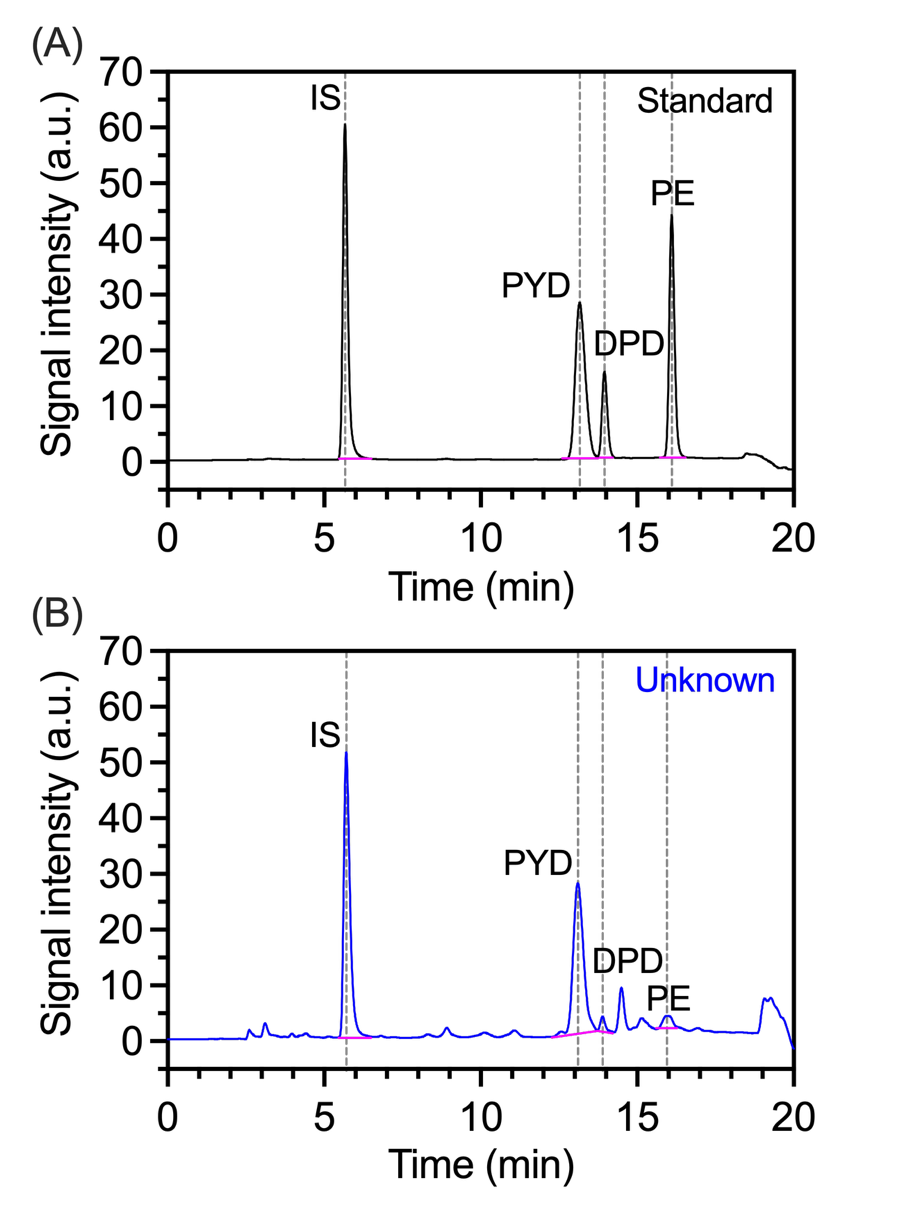


**Figure S3. Examples of the standard (A) and the unknown sample (B) chromatograms from high performance liquid chromatography (HPLC) assays of collagen crosslinks in bone**. Following demineralization of the femurs, the organic matrix was hydrolyzed, lyophilized and reconstituted in the buffer spiked with internal standard (pyridoxine). The HPLC analysis was performed as described under Materials and Methods. Five different standard concentrations were used for calibration of the assay. Areas under the curve (AUC) for each crosslink peak were normalized to AUC of the internal standard. Abbreviation are as follows: IS, internal standard; PYD, pyridinoline; DPD, deoxy-pyridinoline; and PE, pentosidine.
